# Supplementary material for: Deep Sequencing of Immunoglobulin Genes Identifies a Very Low Percentage of Monoclonal B Cells in Primary Cutaneous Marginal Zone Lymphomas with CD30-Positive Hodgkin/Reed–Sternberg-like Cells
Source: Diagnostics (Basel). 2022 Jan 24;12(2):290. doi: 10.3390/diagnostics12020290 (PMC8870847; doi:10.3390/diagnostics12020290)
Supplement: Supplementary file 1 [file diagnostics-12-00290-s001.zip › Supplementary Figures Legend.pdf]

Supplementary Figure Legend

Figure S1. VDJ gene usage and somatic hypermutation analysis of the skin lesions of patient 1. IMGT/V-QUEST and IMGT/Junction analysis output of the IGH nucleotide sequences obtained by NGS (**A**; **B**) showed that the PCMZL with H/RS-like cells and the PCDLBCL shared the same VJ gene segments and an highly hypermutated heavy chain variable gene (IGHV), with 75% and 76% identity to the closest germline IGHV gene respectively (**C**; **D**). They also shared the vast majority of the IGVH somatic hypermutations with only few different mutations (**E**;**F**).

Figure S2. PCR-based analysis of IGK gene rearrangement of the skin lesions of patient 1. The PCMZL with H/RS-like cells and the PCDLBCL showed the presence of a clonal rearrangement of the same size at 289nt in the Vk-Jk master mix and at 284nt in the Vk/Jk Ck intron-Kde master mix. Of note the clonal peaks were smaller in the first biopsy compared to the relapse.
