# Supplementary figures and images for: Deep Sequencing of Immunoglobulin Genes Identifies a Very Low Percentage of Monoclonal B Cells in Primary Cutaneous Marginal Zone Lymphomas with CD30-Positive Hodgkin/Reed–Sternberg-like Cells
Source: Diagnostics (Basel). 2022 Jan 24;12(2):290. doi: 10.3390/diagnostics12020290 (PMC8870847; doi:10.3390/diagnostics12020290)

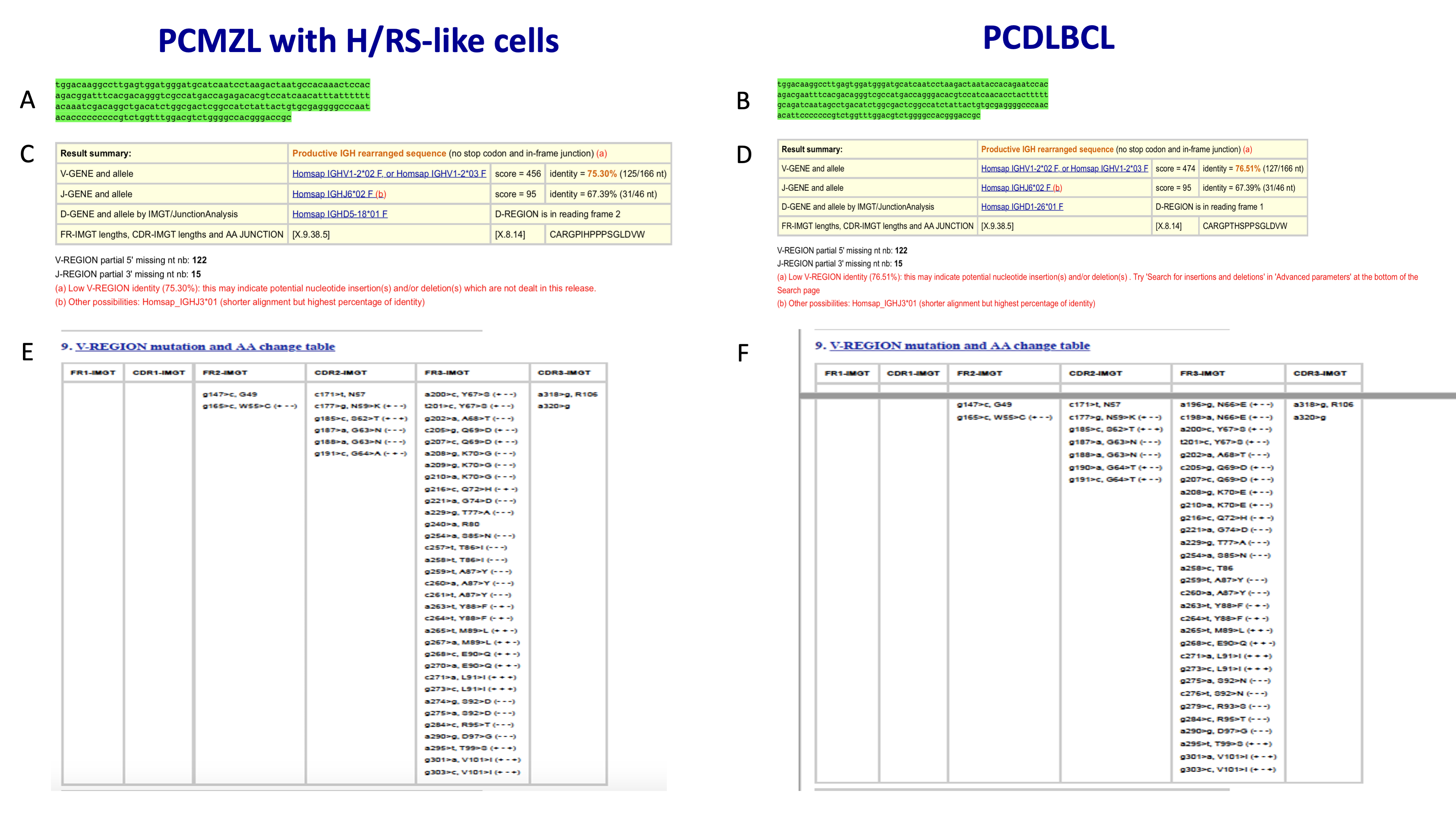

Supplement: Supplementary file 1 [file diagnostics-12-00290-s001.zip › Suppl. Figure S1-SHM case 1.tiff]

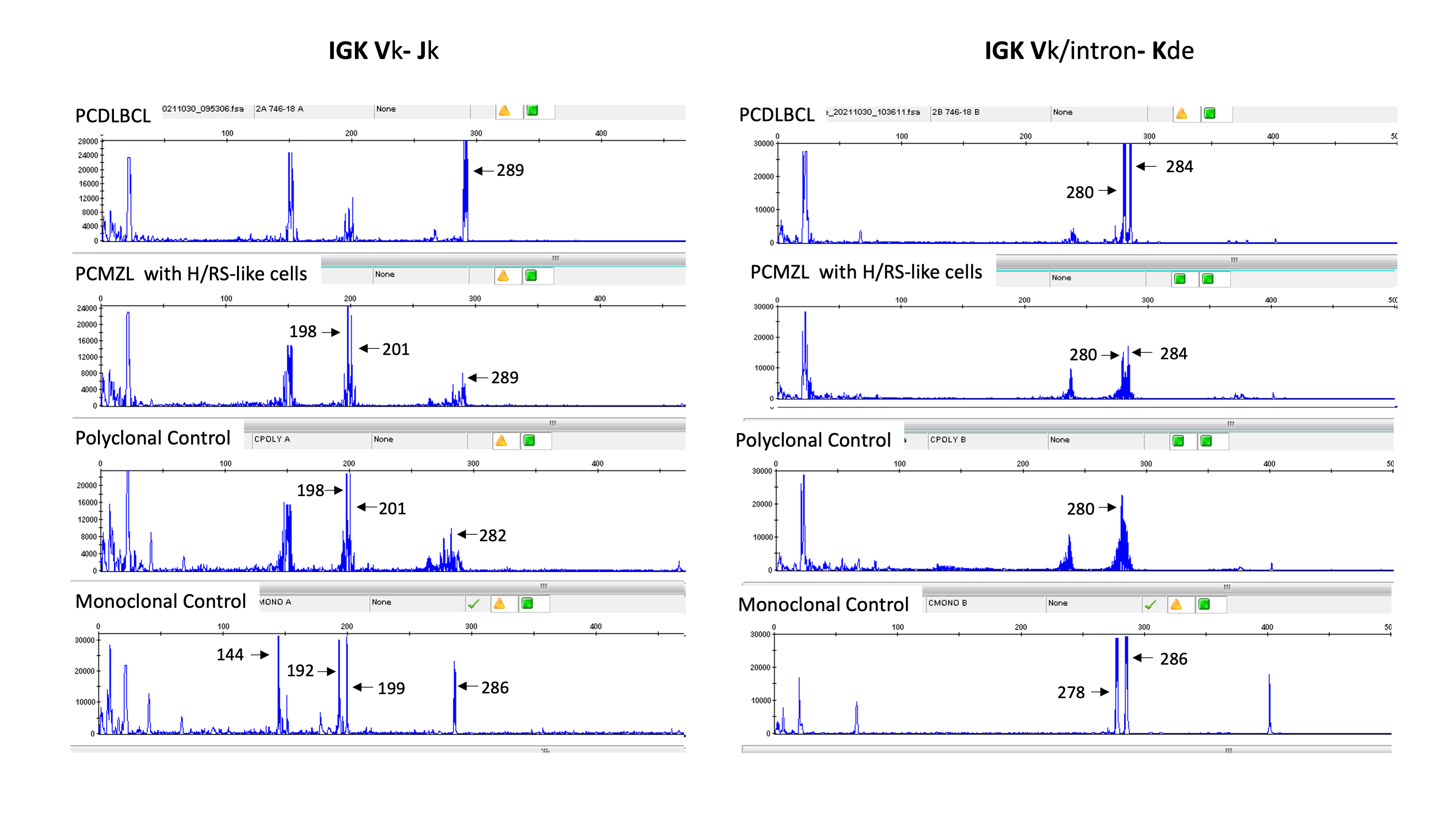

Supplement: Supplementary file 1 [file diagnostics-12-00290-s001.zip › Suppl. Figure S2-IGK case 1.tiff]
